# Retraction: LncRNA FENDRR sensitizes doxorubicin-resistance of osteosarcoma cells through down-regulating ABCB1 and ABCC1n

**DOI:** 10.18632/oncotarget.28908

**Published:** 2026-10-12

**Authors:** Zhu Kun-Peng, Ma Xiao-Long, Zhang Chun-Lin

**Affiliations:** ^1^Department of Orthopaedic Surgery, Shanghai Tenth People’s Hospital Affiliated to Tongji University, Shanghai 200072, PR China; ^2^Institute of Bone Tumor Affiliated to Tongji University School of Medicine, Shanghai 200072, PR China; ^*^These authors contributed equally to this work and co-first authors

**This article has been retracted:** Oncotarget has concluded its investigation of this paper, and the following issues were discovered:

- Figures 2E and 2F: In the colony formation assay, plates MG63/DXR FENDRR-NC and KH/OS si-NC images are duplicates. Additionally, these images were reused in Figure 6B of another paper published by this group [1].
- Figure 5F: The western blot bands for MG63/ABCC1 duplicate those for KH-OS/ABCC1. The ABCB1 bands for FENDRR and si-NC duplicate the KH-OS/DXR-ABCC1 and IL-6 bands for KH-OS/DXR cells. Furthermore, the ABCB1 bands for FENDRR, si-NC, and si-FENDRR were reused in the IL-6 blot for KH-OS/DXR and KH-OS cells. Moreover, the western blots for ABCC3 and GAPDH were found in Figures 3J and 4B of reference [1] as MMP2 and GAPDH, respectively.

Although the authors requested a correction and provided replacement Figures 2 and 5, they did not supply the original files with the datestamps as requested. Furthermore, the corrected version of Figure 5 contains additional duplications that remain unaddressed. Given the number of duplications, the editors can no longer be confident that the overall conclusions remain unaffected. Consequently, the editorial decision has been made to retract this paper. The authors did not agree with the decision.

Original article: Oncotarget. 2017; 8:71881–71893. 71881-71893. https://doi.org/10.18632/oncotarget.17985

## References

[1] Zhu KP, Ma XL, Zhang CL. RETRACTED: LncRNA ODRUL Contributes to Osteosarcoma Progression through the miR-3182/MMP2 Axis. Mol Ther. 2017; 25:2383–93. 10.1016/j.ymthe.2017.06.027. 28750740. Retraction in: Oncotarget. 2025; 33:4008. DOI: 10.1016/j.ymthe.2025.06.030 PMID: 40541189 PMC5628795

